# Long Non-Coding RNA Expression during Aging in the Human Subependymal Zone

**DOI:** 10.3389/fneur.2015.00045

**Published:** 2015-03-09

**Authors:** Guy Barry, Boris Guennewig, Samantha Fung, Dominik Kaczorowski, Cynthia Shannon Weickert

**Affiliations:** ^1^Garvan Institute of Medical Research, Sydney, NSW, Australia; ^2^St Vincent’s Clinical School and School of Biotechnology and Biomolecular Sciences, University of New South Wales, Sydney, NSW, Australia; ^3^Schizophrenia Research Institute, Sydney, NSW, Australia; ^4^Schizophrenia Research Laboratory, Neuroscience Research Australia, Sydney, NSW, Australia; ^5^School of Psychiatry, University of New South Wales, Sydney, NSW, Australia

**Keywords:** long non-coding RNA, subependymal zone, aging, neurodevelopmental disease, neurogenesis, interneuron

## Abstract

The human subependymal zone (SEZ) is debatably a source of newly born neurons throughout life and neurogenesis is a multi-step process requiring distinct transcripts during cell proliferation and early neuronal maturation, along with orchestrated changes in gene expression during cell state/fate transitions. Furthermore, it is becoming increasingly clear that the majority of our genome that results in production of non-protein-coding RNAs plays vital roles in the evolution, development, adaptation, and region-specific function of the human brain. We predicted that some transcripts expressed in the SEZ may be unique to this specialized brain region, and that a comprehensive transcriptomic analysis of this region would aid in defining expression changes during neuronal birth and growth in adult humans. Here, we used deep RNA sequencing of human SEZ tissue during adulthood and aging to characterize the transcriptional landscape with a particular emphasis on long non-coding RNAs (lncRNAs). The data show predicted age-related changes in mRNAs encoding proliferation, progenitor, and inflammatory proteins as well as a unique subset of lncRNAs that are highly expressed in the human SEZ, many of which have unknown functions. Our results suggest the existence of robust proliferative and neuronal differentiation potential in the adult human SEZ and lay the foundation for understanding the involvement of lncRNAs in postnatal neurogenesis and potentially associated neurodevelopmental diseases that emerge after birth.

## Introduction

The human subependymal zone (SEZ; also known as the subventricular zone; SVZ), found adjacent to the lateral ventricle, is the largest reservoir of newly born neuronal and glial cells in the adult brain and, in primates, is estimated to produce ~10 times the number of new neurons compared to hippocampal neurogenesis (1). In late embryonic development, the area lying juxtaposed to the lateral wall of the lateral ventricle and just medial to the ganglionic eminence is responsible for the generation of inhibitory interneurons destined for the cortex and caudate. In adult rodents, proliferative capacity is retained in the SEZ and is critically important to the genesis and replacement of olfactory bulb interneurons that travel from the SEZ via the rostral migratory stream to the olfactory bulb throughout life (2). The extent of SEZ proliferation, neurogenesis, and the existence of a rostral migratory stream is debated in adult humans with some evidence supporting active neurogenesis and migration postnatally, especially in the first few years of human life (3–7). There are also examples of reactive neurogenesis in the human SEZ in response to injury or neural degeneration indicating that this region harbors precursor cells throughout life and implicating activation of this region following brain damage (1). This demonstrates that the adult brain may not only be capable of generating new cells from the SEZ but also that these cells could travel throughout the brain if the necessary instructive cues were present. If the appropriate temporal and spatial requirements of developing cells within the SEZ are not met, altered production of interneuron precursors in the SEZ may also contribute to interneuron deficiencies and the development of brain disorders, such as a host of psychiatric disorders including schizophrenia and autism spectrum disorder (8). There is also evidence that the neurogenic potential of the mammalian brain is attenuated with the normal aging process (9) suggesting that transcriptional events involved in SEZ neurogenesis may also change as humans age.

To more fully understand functional genomic expression in specialized regions of the human brain, such as the SEZ, requires the exploration of the entire transcriptome as the human genome contains only ~1.5% protein-coding sequences (10) but at least 80% of the genome is dynamically transcribed resulting in a transcriptional landscape dominated by long and short non-protein-coding RNAs (11, 12). Long non-coding RNAs (lncRNAs) are expressed from conventional promoters and share many features with protein-coding transcripts, including intron–exon boundaries and alternative splicing; they also commonly possess some distinct features in that many are nuclear-localized and are far more cell- and tissue-specific than protein-coding transcripts (13). There are over 56,000 lncRNAs currently annotated in the human genome (14), many of which have appeared relatively recently in evolution, with about one-third being primate-specific (15).

Long non-coding RNAs display highly specific spatial and temporal expression patterns (13, 16–18), implying that their functions lie in the refinement of regulatory circuits specific to particular cell types and activities, especially the epigenetic control of differentiation and development (19, 20). The majority of lncRNAs are expressed in the brain, many exclusively (13, 15). It is, therefore, unsurprising that they have critical roles in brain development (21) and are emerging as significant contributors to brain disease, such as schizophrenia (22), when dysregulated.

Long non-coding RNAs have been implicated in neurogenesis (23) and functional studies are accumulating demonstrating the mechanisms underpinning their involvement. For example, knockdown studies have identified lncRNAs that are specifically required for neurogenesis, with three lncRNAs (designated N1–N3) shown to be critical for proper neuronal differentiation (24). These lncRNAs also associate with chromatin remodeling complexes, a common theme among lncRNAs such as REST and SUZ12, to control cell fate. Moreover, Ng et al. have demonstrated that RMST, an lncRNA regulated by REST and induced during neurogenesis, interacts with the transcription factor SOX2 and is required for its recruitment to key neurogenesis promoting genes (25). Furthermore, distal-less homeobox 1 (DLX1) while a protein-coding gene, plays an important role in the specification of interneuron subtypes in the SEZ (26, 27), the activity of the sense strand mRNA may be modulated by an antisense lncRNA in mice, termed Dlx1as (28). Although, Dlx1as is thought not to exist in humans (28).

A deeper transcriptomic analysis of human neurogenic regions may be required to determine if there are known and unique lncRNAs expressed in the human SEZ that may be involved in interneuron genesis. Although the SEZ is strongly linked to neurodevelopmental programs and may be a source of adult neurogenesis, there has been no study to date investigating the non-coding RNA molecular landscape of this region and transcriptional changes during aging. Therefore, we sought to characterize age-dependent expression of protein-coding RNA and lncRNA transcripts to gain insight into SEZ neurogenesis.

## Materials and Methods

### Human subependymal zone tissue

Tissue from the anterior caudate of 21 normal individuals between the ages of 21 and 81 years was obtained from the New South Wales Tissue Resource Centre (Sydney, NSW, Australia; HREC 07261). Cases were screened for neuropathology and toxicology and were free of psychiatric or neurological disease. The cause of death for most cases was cardiac (cardiac *n* = 17, cardiac/respiratory *n* = 1, unknown *n* = 2, cancer *n* = 1) and the cohort consisted of 3 females and 18 males, with an average age of 53.95 years, average PMI of 30.1, average pH of 6.68, and average RIN of 7.3 (Table S1 in Supplementary Material). Fresh-frozen caudate tissue was sectioned on a Leica CM3050 S cryostat, taking sets of 20μm × 60 μm sections interspersed with 10μm × 14 μm sections. Tissue for RNA extraction was dissected from 60 μm thick sections while frozen. Cuts were made ~2 mm deep to the lateral surface of the lateral ventricle to include the SEZ using Wescott spring scissors (T106, ProSci Tech). For each case, tissue was dissected from 3 sets of 3–4 adjacent 60 μm sections spaced ~1340 μm to give 10 sections/case (~40 mg tissue total/case).

#### RNA extraction

Total RNA was extracted from SEZ-containing tissue for each case using Trizol (Invitrogen), and RNA quality was assessed on an Agilent Technologies 2100 Bioanalyzer with an RNA 6000 Nano kit (Agilent Technologies, USA) according to the manufacturer’s instructions. cDNA was synthesized from 3 μg total RNA per case using SuperScript^®^ III First-Strand Synthesis kit and random hexamers (Invitrogen). For LINC00657, NEAT1, Dlx1, and Dlx1 enhancer, 2 μg of total RNA was DNase treated using TURBO DNA-free kit (Ambion) prior to cDNA synthesis to minimize genomic DNA amplification.

### Quantitative real-time PCR

#### TaqMan gene expression assays

Transcript levels were measured by quantitative real-time PCR (qPCR) on an ABI Prism 7900HT Fast Real-time PCR system with 384-well format and TaqMan Gene Expression Assay [Applied Biosystems; Ki67, Hs010324433; see Ref. (29)]. Changes in expression of mRNAs for one coding (DCX) and two expressed lncRNAs, one novel (LINC00657) and one known (NEAT1), with age were validated with qPCR in the entire cohort with a total of 21 cases (Table S1 in Supplementary Material). TaqMan gene expression assays Hs01035496_m1 and Hs01008264_s1 were used to detect DCX and NEAT1 mRNAs, respectively, and custom probe AI6RO2N was used to detect LINC00657. All measurements from each subject were performed in duplicate and relative quantities determined from a seven-point standard curve of pooled cDNA. Transcript quantities for Ki67 were normalized by the geometric mean of four housekeeping genes: ubiquitin C (Hs00824723_m1), actin β (Hs99999903_m1), glyceraldehyde-3-phosphate dehydrogenase (Hs99999905_m1), TATA box binding protein (Hs00427620_m1) that did not correlate with age (*r* = −0.02, *p* > 0.05). Quantity means for DCX, LINC00657, and NEAT1 were normalized to glyceraldehyde-3-phosphate dehydrogenase (Hs99999905_m1) transcript expression.

#### Sybr green

Quantitative real-time PCR was also conducted using KAPA SYBR Fast qPCR universal kit (KAPA Biosystems, USA) according to manufacturer’s instructions in 384-well plates. Primers used were DLX1 (forward: CTCAGGTCAAGATCTGGTTC; reverse: GGATGAAGAGTTAGGGTTCC), DLX1 enhancer (forward: CGAGGATTAACACTTCCTGAA; reverse: GGGAGTGATTATGTATGCACC), and GAPDH (forward: CAGCCTCAAGATCATCAGCA; reverse: ATGGACTGTGGTCATGAGTC). Ten microliter qPCR reactions were performed in triplicate using 2 μL of diluted cDNA per reaction. Quantitative PCR reagent master mixes included 2× KAPA SYBR FAST qPCR Master Mix (2×) Universal, ROX-high reference dye and 200 nM final concentration of each forward and reverse primer (Integrated DNA Technologies, Inc., USA). An Applied Biosystems 7900HT Fast Real-Time PCR machine fitted with a 384-well thermal block was used for qPCR (Life Technologies, USA). Thermal cycling conditions consisted of initial 3 min enzyme activation at 95°C followed by 40 cycles of 95°C for 5 s, 60°C for 30 s, and finished with the default instrument dissociation protocol. Melt curve (dissociation) analysis was performed to identify the presence of primer–dimers and to analyze the specificity of the reaction. Amplicon sizes were verified for all primer pairs by agarose gel electrophoresis. Initial analysis of raw qPCR data utilized SDS v2.4.1 software (Life Technologies, USA).

### Deep sequencing

Eleven samples were chosen for deep sequencing based on cost limitations while maintaining statistical relevance and the best possible spread of age and sex.

#### RNA isolation for library preparation

Prior to library preparation, 1.5 μg of each total RNA sample was DNase-treated using TURBO DNase (Ambion, USA) according to the manufacturer’s instructions followed by purification with Agencourt RNAClean XP beads (Beckman Coulter, USA) also according to manufacturer’s instructions. RNA concentration was measured using a Nanodrop 2000 spectrophotometer (Thermo Fisher Scientific, USA).

#### Library preparation

Five hundred nanograms of total RNA were used as input material for library preparation using the TruSeq Stranded Total RNA Sample Prep Kit (Illumina, USA) according to manufacturer’s instructions including the recommended modification to the RNA fragmentation duration to account for partially degraded RNA. Individual libraries were indexed as recommended by Illumina.

#### Quantification and quality control of DNA library templates

Indexed DNA libraries were analyzed individually using an Agilent Technologies 2100 Bioanalyzer with the DNA 1000 kit according to the manufacturer’s instructions (Agilent Technologies, USA). Libraries were diluted and pooled to a final concentration of 10 nM each in nuclease-free H_2_O (Ambion, USA). Pooled libraries were quantitated using a Life Technologies Qubit 2.0 Fluorometer with the Qubit dsDNA HS Assay Kit (Life Technologies, USA) and further diluted to 2 nM. Final DNA library concentration was confirmed using a Qubit dsDNA HS Assay Kit. PCR-competent library DNA concentration was verified using the universal KAPA Library Quantification Kit for Illumina Sequencing Platforms according to manufacturer’s instructions (KAPA Biosystems, USA).

#### Sequencing

Total RNA sequencing was performed using the Illumina HiSeq2500 platform with 100 bp paired-end sequencing with a fragment size of ~295 bp. Illumina TruSeq version 3 chemistry was used for cluster generation and sequencing.

### Bioinformatic analysis

Initial and post trimming quality control was performed with FastQC (version 0.10.1). Adapter and quality trimming was performed with TrimGalore [version 0.3.3; (30)] including adapter cutting, a minimal length of 20 bp and a Quality Phred score cutoff of 20. Trimmed paired-end reads were aligned against assembly GRCh37 of the human genome with TopHat [version 2.0.10; (31)] and Bowtie [version 2.1.0; (32)] using a pre-built transcriptome based on Gencode (version 19). Post processing and quality control of the alignment was performed with Bedtools [version 2.17.0; (33)], Samtools [version 0.1.19; (34)], Samstat [version 1.08; (35)], and RNA-SeQC [version 1.1.7; (36)]. The pipeline incorporating the aforementioned algorithms was built with NGSane [version 0.4.0; (37)]. Transcript normalization was performed using upper quartile normalization applied through the edgeR package (38). The average million reads per sample are ~16 million paired-end reads (16,106,463) and the average mapping of these reads is 94.38%. For transcriptome analysis, alignment counts were resolved on gene and transcript identifiers based on Gencode (version 19) annotation. Quantification was performed post alignment with HTSeq (version 0.5.4p5) in union mode considering only uniquely mapped reads. Transcriptome assembly was carried out with Cufflinks (39). Age regression analysis was performed using GraphPad Prism Version 6.0B with a linear regression fit and a 95% confidence interval with a *p* value of <0.05 deemed significant.

## Results

### Genome-wide transcriptomic analysis of the adult human SEZ

**Figure 1:**
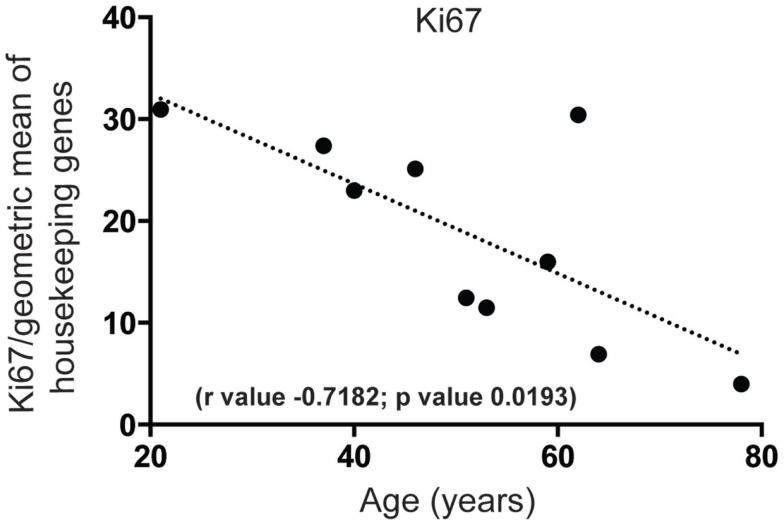
**Proliferation significantly decreases in the human SEZ during aging**. Quantitative PCR reveals that mRNA for the proliferation marker Ki67 sharply decreases in an age-dependent manner. (Significance is defined as *p* value <0.05).

**Figure 2:**
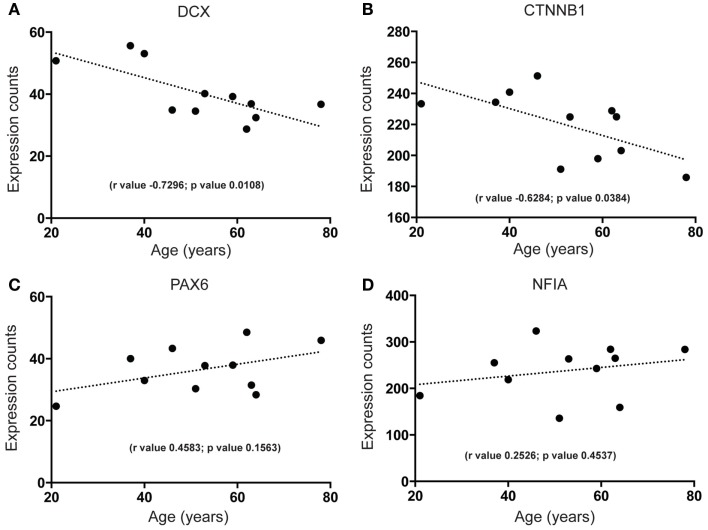
**Next generation sequencing shows age-dependent decreases in early neuron differentiation and putative proliferation markers but stable expression of neural progenitor markers in the human SEZ**. Significant decreases during aging are observed in the expression levels of **(A)** the immature neuronal marker doublecortin (DCX; validated by quantitative PCR; Figure S2A in Supplementary Material) and **(B)** a key promoter of neuronal proliferation β-catenin (CTNNB1). Non-significant age-dependent alterations are observed in the expression of radial glial markers **(C)** paired box 6 (PAX6) and **(D)** nuclear factor I A (NFIA). (Significance is defined as *p* value <0.05).

First, we wanted to confirm that proliferating cells within the cell cycle are likely to exist in the human SEZ. By qPCR we found that mRNA for the proliferation marker Ki67, required in all active phases of cell division while absent in the non-proliferating state, was present and that the levels of this transcript decreased sharply with increasing age (Figure 1). Next, to determine the molecular identity of lncRNAs in the adult human SEZ and to understand the mechanisms underpinning neurogenesis that may be applicable during adulthood, we used next generation sequencing (NGS) for genome-wide analysis. Transcript expression levels were ranked according to average HTSeq counts (expression counts; Table S2 and Figure S1 in Supplementary Material) over the 11 samples. Interrogating the top 100 expressed transcripts using DAVID 6.7 analysis for functional annotation (40) revealed highly significant cluster annotation (high stringency) for neuron development and differentiation (Enrichment score 6.18; Table S3 in Supplementary Material) providing confidence that this dataset may be valuable for investigating neurogenesis-related mechanisms in the human SEZ and supporting that this region is potentially enriched in immature neurons. We confirmed a decrease in neuronal differentiation in the human SEZ with advancing age as our NGS data revealed significant declines in the early neuronal differentiation and migration marker doublecortin [DCX; (41); Figure 2A; confirmed by qPCR (Figure S2A in Supplementary Material) in a larger cohort of samples (Table S1 in Supplementary Material)] and predicting additional mRNAs, which can be involved in proliferation, such as β-catenin [CTNNB1; (42); Figure 2B]. Interestingly, however, the neuronal progenitor marker paired box 6 [PAX6; (43); Figure 2C] and the early glial specification factor nuclear factor I A [NFIA; (44); Figure 2D], were predicted to be not significantly altered with age and trended upwards with increasing age implying that the progenitor pool for neurogenesis may persist during aging. These data suggest that significant neurogenesis may still be possible in the adult brain but proliferative levels of certain precursor cells may decline with regards to age.

### Increase in inflammatory markers in the human SEZ during aging

**Figure 3:**
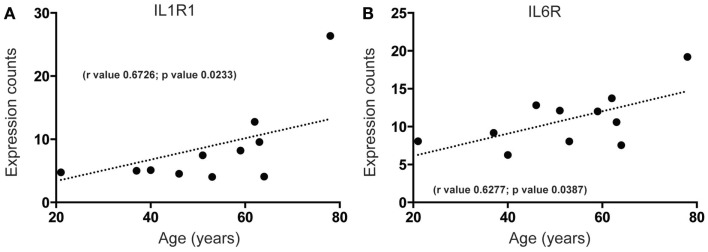
**Inflammatory receptor mRNAs related to senescence increase in the human SEZ with age**. Significant increases during aging are observed in the expression levels of **(A)** interleukin-1 receptor 1 (IL1R1) and **(B)** interleukin-6 receptor (IL6R). (Significance is defined as *p* value <0.05).

Increased brain inflammation with aging is thought to underlie, at least in part, the gradual decline of human brain function (45, 46). Our data predict that mRNA-encoding receptors of inflammatory mediators interleukin (IL)-1 and IL-6, IL1R, and IL6R, are significantly upregulated during aging in the human SEZ (Figures 3A,B). These changes were in contrast to the predicted decrease in proliferative (Figures 1 and 2B), progenitor (Figures 2C,D), and immature neuron (Figure 2A) markers, and could reflect indicators of brain injury, senescence, or neurogenic impairment. However, as our samples were from donors with no overt phenotype these increases are likely due to the involvement of IL-1 and IL-6 pathways in “normative” senescence (47).

### A subset of lncRNAs are highly expressed and upregulated during aging in the human SEZ

**Figure 4:**
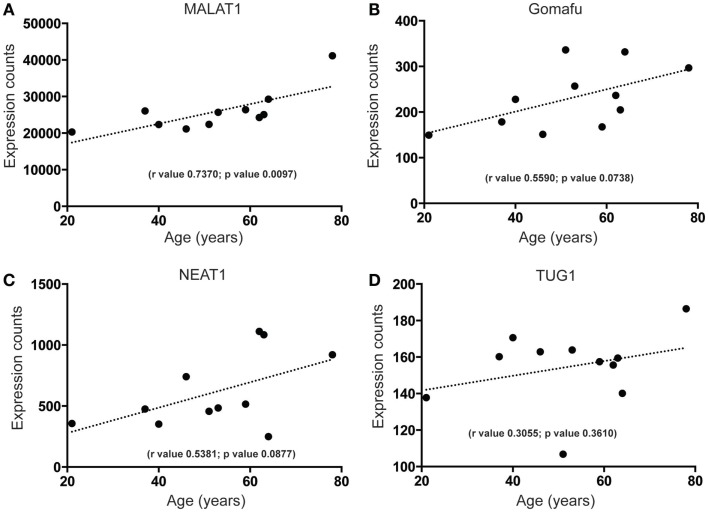
**Highly expressed lncRNAs that increase in expression during aging in the human SEZ**. A significant increase during aging in the human SEZ is observed in the expression levels of **(A)** MALAT1. Insignificant, although upward trending, increases in expression during aging are also seen for the highly expressed lncRNAs **(B)** Gomafu, **(C)** NEAT1, and **(D)** TUG1. (Significance is defined as *p* value <0.05).

To begin to characterize more fully the molecular signature of lncRNAs in the human SEZ, we determined the highest expressed lncRNAs from the human SEZ NGS data (Table 1). Expectedly, due to the generally low expression of lncRNAs in any particular brain region, we found that only 30 lncRNAs fell into the top 2262 of the most highly expressed transcripts, i.e., the 30th most highly expressed lncRNA was 2262nd (Table S2 in Supplementary Material). We chose the top 30 as they represent a reasonably high level of expression. The fairly well-known, widely and robustly expressed lncRNAs, such as MALAT1, involved in neuronal function (48) and proliferation (49), and NEAT1, implicated in paraspeckle formation and alternative splicing (50), were among the most highly expressed lncRNAs in the human SEZ. Additional lncRNAs that have been linked to differentiation were in the highly expressed group (Table 1) and were predicted to increase in expression during aging [Figures 4A–D; NEAT1 examined by qPCR increases with age; however, the relationship is not significant (Figure S2B in Supplementary Material) in a larger cohort of samples (Table S1 in Supplementary Material)]. For example, the lncRNA Gomafu is implicated in neuronal differentiation (18) and alternative splicing in human cortical neurons (22) and we find that Gomafu expression is suggested to increase in the human SEZ during aging. This is also true for the lncRNA TUG1 that is a direct target of the tumor suppressor p53 and reported to regulate proliferation (51).

**Table 1:** **Most highly expressed lncRNAs in the human SEZ**.

ENGS_ID	Genes	Average (counts)	SD
ENSG00000251562.3	MALAT1	25823.83865	5448.001106
ENSG00000258486.2	RN7SL1	7552.087429	4836.752255
ENSG00000259001.2	RPPH1	3233.617342	1543.386952
ENSG00000269900.2	RMRP	1379.439891	282.2752341
ENSG00000214548.10	MEG3	1244.18418	266.2397648
ENSG00000236824.1	BCYRN1	1104.320094	299.1965189
ENSG00000260032.1	LINC00657	1103.68697	259.3204245
ENSG00000245532.4	NEAT1	613.6523589	210.4050131
ENSG00000247556.2	OIP5-AS1	485.6564217	71.60747981
ENSG00000269821.1	KCNQ1OT1	454.9719056	64.03659426
ENSG00000229807.5	XIST	426.524232	495.9491165
ENSG00000257151.1	RP11-701H24.2	343.6736379	77.53051621
ENSG00000249614.1	RP11-703G6.1	231.6482767	58.90302218
ENSG00000225783.2	MIAT	230.6506684	56.30339301
ENSG00000259380.1	RP11-346D14.1	228.4430582	51.89955342
ENSG00000263934.2	SNORD3A	216.0758427	51.21067197
ENSG00000239002.2	SCARNA10	209.7943427	24.60247805
ENSG00000242808.3	SOX2-OT	200.6236003	62.92766253
ENSG00000249348.1	UGDH-AS1	194.0595263	35.73419584
ENSG00000232164.1	AC092669.3	180.1620377	35.64551905
ENSG00000258441.1	LINC00641	165.1920813	32.95864173
ENSG00000253352.4	TUG1	154.6567408	17.20914581
ENSG00000255794.2	RMST	147.9867556	36.22319234
ENSG00000260918.1	RP11-731J8.2	143.6049122	33.63902851
ENSG00000263244.1	RP11-473I1.10	141.0085938	19.45147484
ENSG00000231074.4	HCG18	140.0152075	13.24767675
ENSG00000225733.1	FGD5-AS1	139.7240432	18.8825367
ENSG00000255248.2	RP11-166D19.1	117.5159698	29.70264099
ENSG00000250366.2	LINC00617	88.79670147	16.83608733
ENSG00000242125.2	SNHG3	87.05553931	13.80532119

### A unique subset of lncRNAs of unknown function decreased during aging in the human SEZ

**Figure 5:**
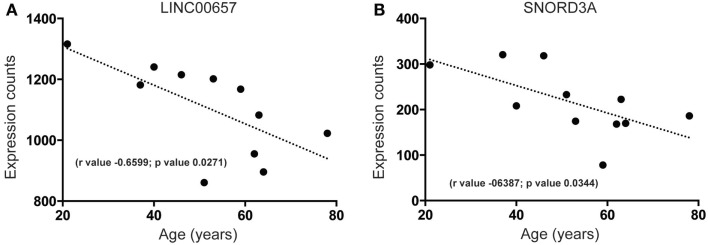
**Examples of highly expressed and uncharacterized lncRNAs that are downregulated during aging in the human SEZ**. Significant age-related decreases in the human SEZ are observed for the expression of the lncRNAs **(A)** LINC00657 (Validated by quantitative PCR; Figure S2C in Supplementary Material) and **(B)** SNORD3A. (Significance is defined as *p* value <0.05).

Our analysis of the SEZ reveals that there are highly expressed and uncharacterized lncRNAs that display significant regulation that parallel decreased age-related proliferation such as LINC00657 (Figure 5A) and SNORD3A (Figure 5B). The function of LINC00657 is unknown but this lncRNA appears to decline rapidly during aging (Figure 5A), a result validated through qPCR in a larger cohort of samples (see Figure S2C and Table S1 in Supplementary Material). This lncRNA is conserved in vertebrates (Figure S3 in Supplementary Material) and, although widely expressed, is enriched in the brain (Figure S3 in Supplementary Material). SNORD3A, an lncRNA encoding a small nucleolar RNA (C/D Box 3A), is predicted to be similarly downregulated during aging in the human SEZ (Figure 5B). This lncRNA is mammalian-specific (Figure S4 in Supplementary Material) and its expression levels are lower in brain tissue than elsewhere (Figure S4 in Supplementary Material) and may reflect expression specificity in particular cell types or regions, or dependence on activity in the human brain.

### Possible enhancer transcript for DLX1 detected in the human SEZ

**Figure 6:**
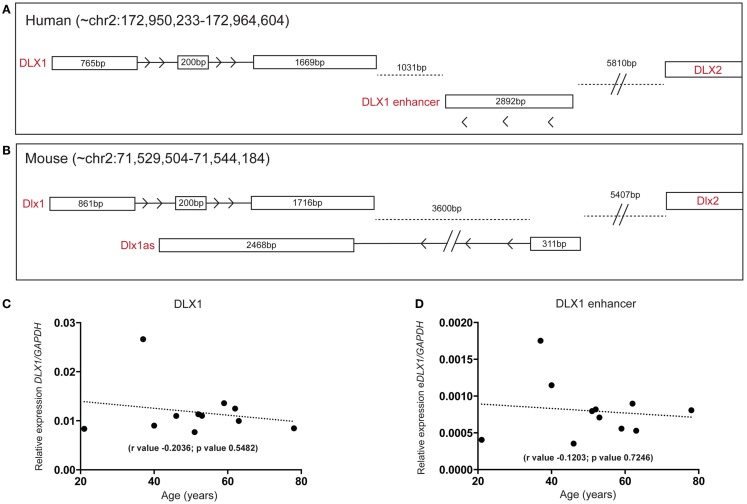
**Detection of a human DLX1 antisense transcript that may function as an enhancer in the human SEZ**. Graphical depiction of the corresponding **(A)** human (DLX1, antisense transcripts, and DLX2) and **(B)** mouse (Dlx1, Dlx1as, and Dlx2) regions of the genome. Both **(C)** DLX1 and **(D)** the putative DLX1 enhancer transcript expression are predicted to remain relatively constant during aging in the human SEZ. (Significance is defined as *p* value <0.05).

A lncRNA, antisense to the mouse Dlx1 gene (Dlx1as), may regulate Dlx1 function and hence interneuron specification but is proposed not to exist in humans (28). Interestingly, when we examined our human SEZ RNAseq data specifically examining the DLX locus, our analysis revealed reads that aligned to the syntenic region of Dlx1as. The position and structure of the human antisense transcript appears altered compared with mouse (Figures 6A,B). The total size of the antisense transcripts (excluding intronic regions) between mouse and human are comparable; however, the human transcript seems to have lost the DLX1-overlapping portion and may not possess an intron as seen in the mouse (at least in the adult human SEZ transcript pool). In a comprehensive study of enhancer regions, Andersson and colleagues designated this transcript as a possible actively transcribed enhancer (52). The putative human enhancer DLX1 (eDLX1) transcript is expressed at much lower levels in the SEZ than DLX1 (Figure 6C) but is of interest as neither ENCODE/GENCODE, RefSeq, UCSC (Figure S5 in Supplementary Material) nor the Illumina Body Atlas (data not shown) could find evidence of the human transcript emanating from the opposite DNA from the DLX1 sense strand. This may be due to the fact that it is uniquely expressed in the SEZ, or other discrete regions, and no other study has sequenced this region in depth. It remains to be seen whether this transcript indeed acts as an enhancer or traditional antisense regulator. Both DLX1 (Figure 6C) and eDLX1 (Figure 6D) are predicted to be expressed at relatively stable levels throughout the age range studied.

## Discussion

In the case of neurodevelopmental diseases, interneuron maldevelopment may underlie conditions such as schizophrenia and autism due to the fact that interneurons are specified and still developing well into postnatal life (7, 53–55) and accumulating evidence supports an inhibitory neuron dysfunction that may be common in both conditions and is, in fact, well replicated in schizophrenia (8, 56, 57). However, the role of interneurons in brain disorders first diagnosed in older adults or in normal brain aging is not as clear. Although different types of interneurons are present in the brain, a general deficit in interneuron progenitor pools could affect all interneuron subtypes or it may be that interneuronal specification potential changes with age such that abnormal molecular environments arising at a particular life stage could lead to distinct outcomes. As the human SEZ is a major source of interneuron production during development (1) and may contribute to adult neurogenesis, an understanding of the molecular control of this region is fundamental to our understanding of human brain diseases. In this study, we provide transcriptional evidence to suggest that interneuronal genesis may continue throughout human life in the SEZ, including the detection of mRNA encoding a molecule (DCX) known to be expressed by migrating interneurons (6, 58–60), and an mRNA encoding a transcription factor capable of directing cell fate toward the interneuron phenotype, DLX, and finally a putative DLX1 enhancer transcript, perhaps providing a window into unique transcripts that operate to induce proliferation, cell differentiation, and early neuronal migration even into advanced years of life in humans.

Our transcriptomic analysis of the human SEZ during adulthood revealed a bias for neuronal differentiation and an lncRNA “signature” that includes a small subset of highly expressed functionally known and unknown lncRNA transcripts. Interestingly, our data predict that the expression of some putative proliferative mRNA markers (Ki67, CNTTB1) and immature neuron markers (DCX) decrease during aging but intriguingly, progenitor markers (PAX6, NFIA) remain stable suggesting that the transition from multipotent progenitor cells to more committed precursors and neuroblasts (those in a transitional state) is reduced with age. An age-dependent decrease in neurogenesis seemed to coincide with the predicted increase in some of the highly expressed and proliferation-associated lncRNAs, such as Gomafu, MALAT1, and NEAT1 that form nuclear speckles and require activity to perform their functions. We did not anticipate this result, and it may be that during aging these lncRNAs become unresponsive to mitogenic or environmental stimulation or the stimuli themselves are diminished or not relayed correctly.

Several studies suggest that genesis of new neurons may be induced in the adult brain under trauma such as ischemia and injury (61). Injury or disease resulting in inflammation increases levels of pro-inflammatory cytokines such as IL-6, and we have previously reported that increased cortical cytokines are related to increased density of neurons in the white matter in adult humans, which can be interpreted as a possible increase in immature migratory neurons (56, 59). Furthermore, in this study, we predicted significant increases in IL1R and IL6R in the human SEZ with advancing age (Figure 3). These observations coupled with other reports of increased pro-inflammatory cytokines in brain aging (62) would suggest that we may expect to find increases in cell proliferation and early neuronal differentiation in the aging SEZ. However, this was not the case. While it may be that increases in inflammatory cytokines can be involved with inducing neurogenesis in a younger brain (63), inflammatory pathways are also implicated in senescence (47) and IL-6 can inhibit neuronal differentiation of neural stem cells (64) and astrocytic overexpression of IL-6 inhibits hippocampal neurogenesis (65). We find that the mRNA-encoding cytokine receptors for both IL1 and IL6 are suggested to increase in the aging human SEZ while the proliferation marker Ki67 is decreased. Our results would be consistent with a speculative model where receptors for cytokines may be involved in sensing and translating the demise of the human brain and stimulating senescence of the SEZ. It is also possible that although inflammation-induced neurogenesis is potentially viable in the young brain this system may become more sensitized to inflammation in the aging brain making it unable to respond to injury- or degeneration-induced inflammation in the same manner (66).

### Future directions

Our data suggest that neurogenesis in the adult human brain continues across the typical life span. However, normal levels of neurogenesis may be hampered in aging perhaps by an altered ability of resident SEZ progenitor cells to successfully initiate proliferation in response to extrinsic cues, i.e., up regulation of cytokines. Additionally, we have identified that many lncRNAs are expressed in this region, some of which may be unique. Future experiments aimed at a greater understanding of the role of lncRNAs in human neurogenesis and how cell intrinsic molecular factors may interact with cell extrinsic molecular cues in cell specific patterns over aging are needed to advance our understanding of how to harness and control new neuronal birth. Also, separating these possibilities of change in proliferative response of neuronal precursors to cytokines over aging would be of great relevance to the field. Furthermore, mechanistic insight into the function of highly expressed and uncharacterized lncRNAs in the human SEZ could uncover neurogenic regulatory processes central to this region. We believe that this study will inform future analyses of neurodevelopmental and neurodegenerative diseases and highlight the need to examine lncRNAs in larger sample sizes in future transcriptomic studies to reveal region-specific function.

## Conflict of Interest Statement

Cynthia Shannon Weickert is a member of the advisory board for Lundbeck and consultant for Roche Pharmaceuticals. The other co-authors declare no conflict of interest.

## Supplementary Material

The Supplementary Material for this article can be found online at http://www.frontiersin.org/Journal/10.3389/fneur.2015.00045/abstract
