# Supplementary material for: Long Non-Coding RNA Expression during Aging in the Human Subependymal Zone
Source: Front Neurol. 2015 Mar 9;6:45. doi: 10.3389/fneur.2015.00045 (PMC4353253; doi:10.3389/fneur.2015.00045)
Supplement: Supplementary file 1 [file Image_1.PDF]

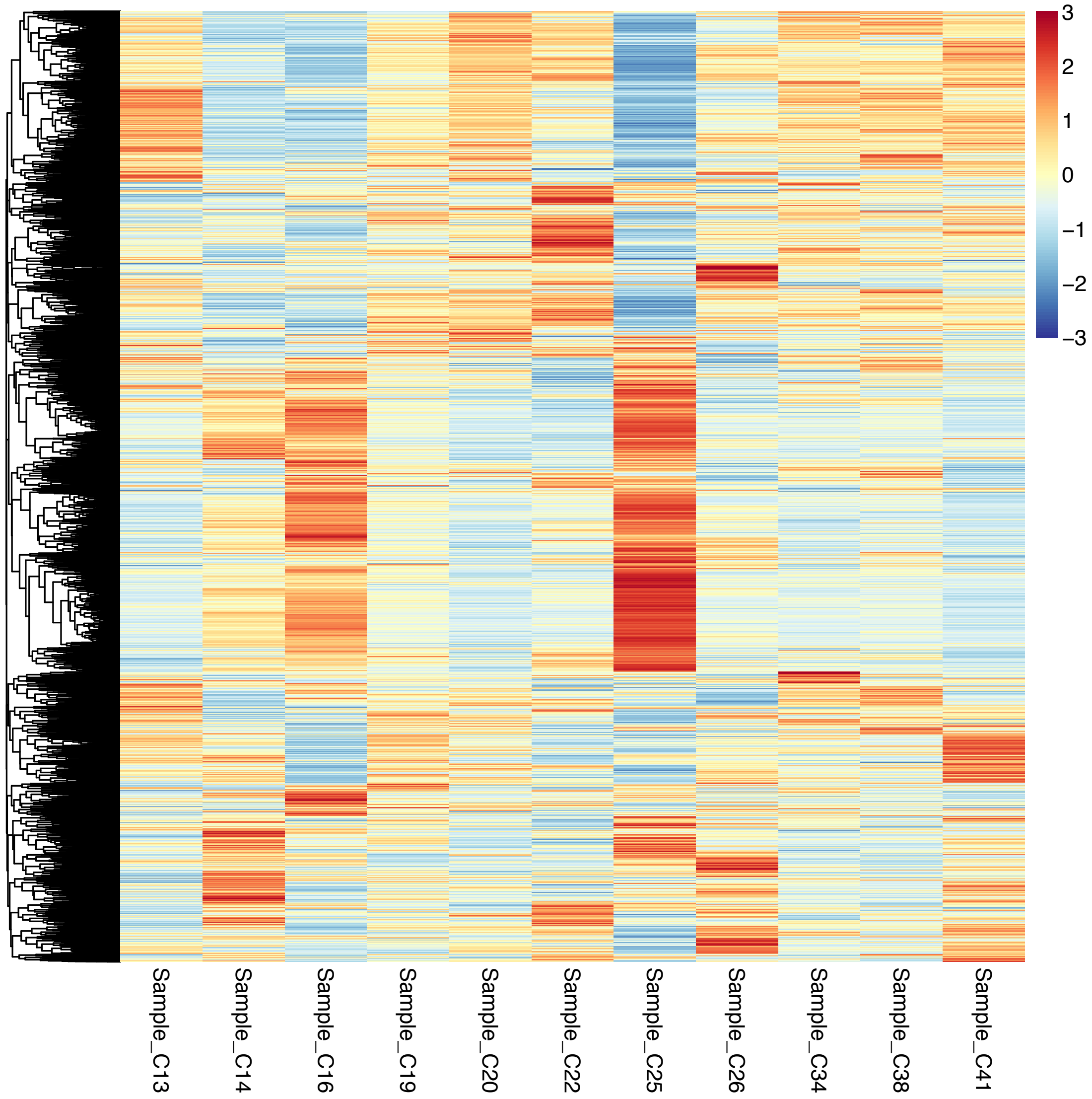

Supplementary Figure 1: Heatmap and unsupervised clustering of all expressed transcripts in the samples following next generation sequencing. Specific transcript values, either upregulated (red) or downregulated (blue), in comparison to the mean of all the samples.
