# Supplementary material for: Long Non-Coding RNA Expression during Aging in the Human Subependymal Zone
Source: Front Neurol. 2015 Mar 9;6:45. doi: 10.3389/fneur.2015.00045 (PMC4353253; doi:10.3389/fneur.2015.00045)
Supplement: Supplementary file 2 [file Image_2.PDF]

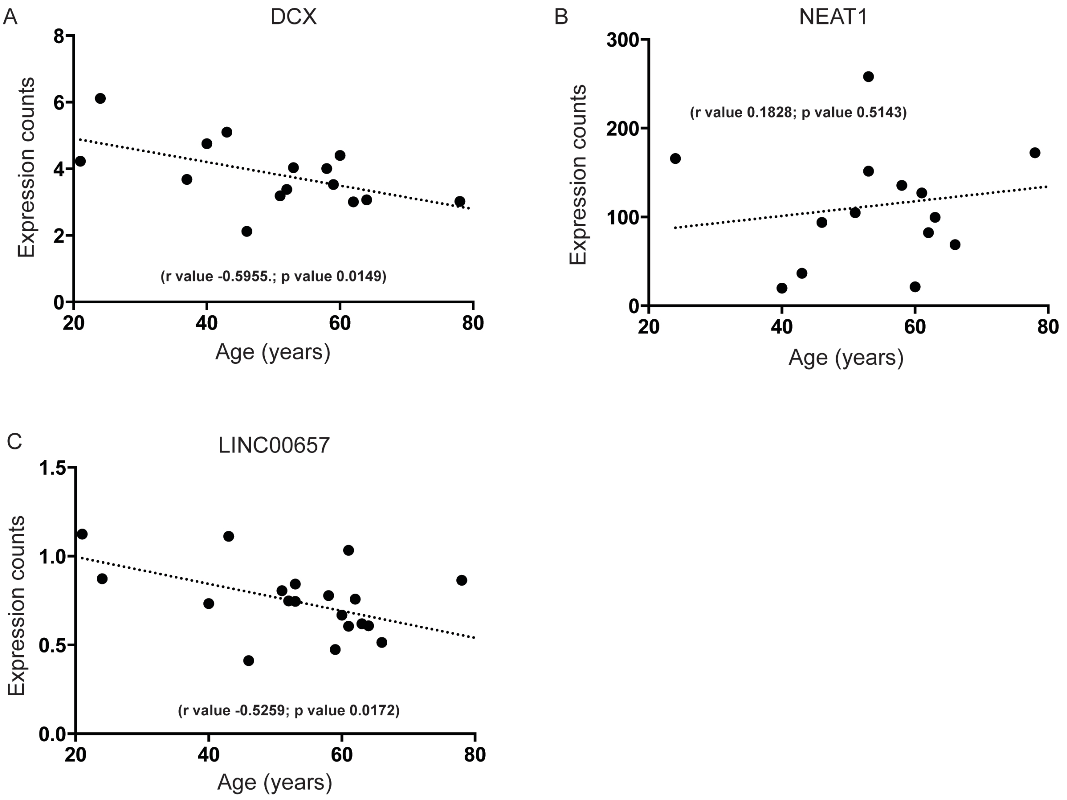

Supplementary Figure 2: Quantitative PCR (qPCR) validation of next generation sequencing analysis. Reductions in both DCX (A) and LINC00657 (C) mRNAs with age were replicated by qPCR in an extended cohort. NEAT1 mRNA expression by qPCR was not significantly correlated with age, however shows a non-significant increase with age (B).
