# Supplementary material for: Long Non-Coding RNA Expression during Aging in the Human Subependymal Zone
Source: Front Neurol. 2015 Mar 9;6:45. doi: 10.3389/fneur.2015.00045 (PMC4353253; doi:10.3389/fneur.2015.00045)
Supplement: Supplementary file 3 [file Image_3.PDF]

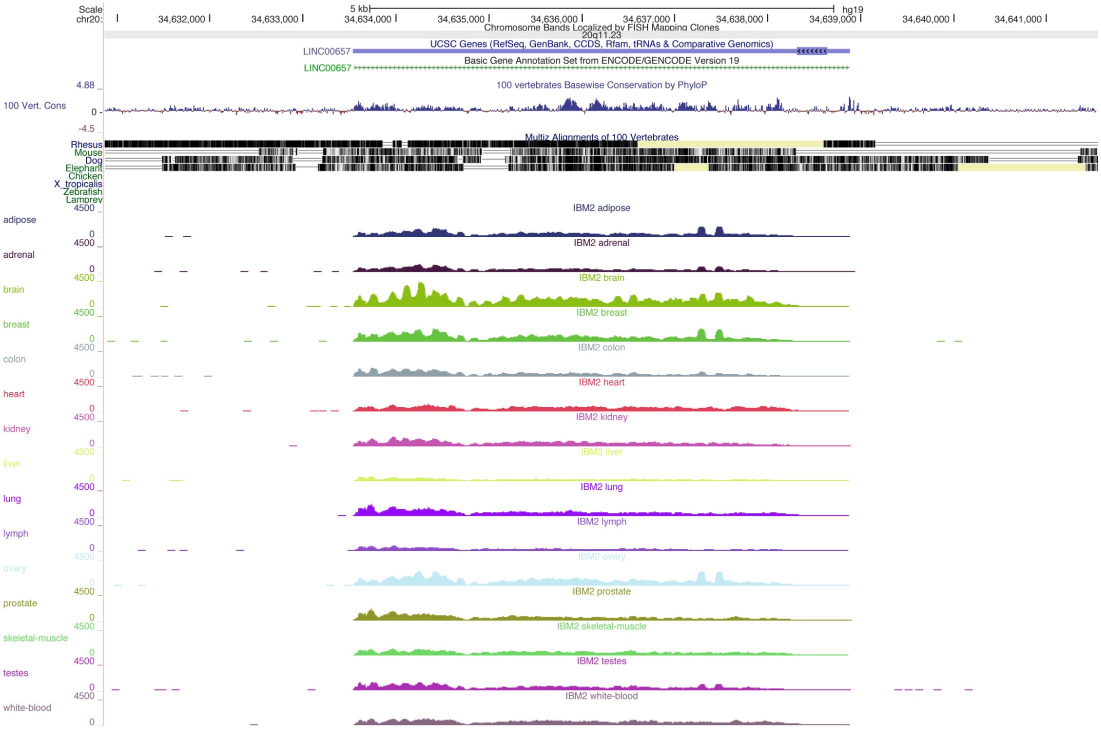

Supplementary Figure 3: Snapshot of the UCSC genome browser (HG19) showing the LINC00657 locus. LINC00657 seems to be well conserved in mammals (see 100 Vertebrate Conservation track) and its expression is ubiquitous but higher in brain (Illumina Body Atlas tracks; 15 human tissues).
