# Supplementary material for: Long Non-Coding RNA Expression during Aging in the Human Subependymal Zone
Source: Front Neurol. 2015 Mar 9;6:45. doi: 10.3389/fneur.2015.00045 (PMC4353253; doi:10.3389/fneur.2015.00045)
Supplement: Supplementary file 4 [file Image_4.PDF]

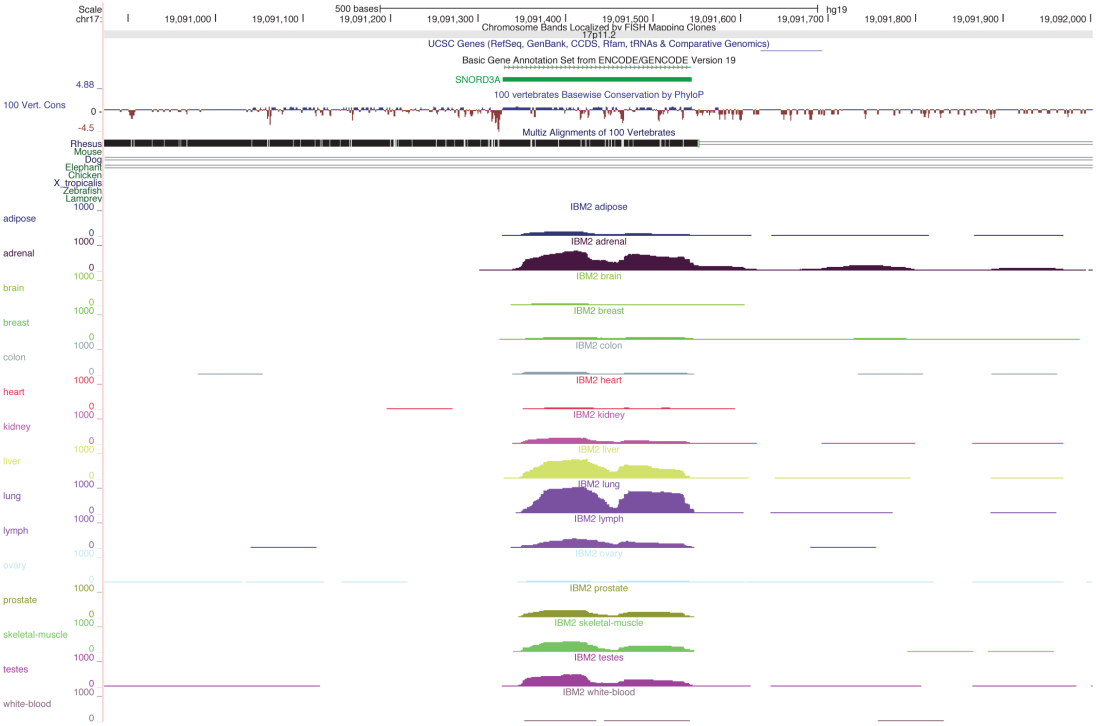

Supplementary Figure 4: Snapshot of the UCSC genome browser (HG19) showing the SNORD3A locus. SNORD3A seems to be primate-specific (see 100 Vertebrate Conservation track) and its expression is ubiquitous with lower levels observed in brain (Illumina Body Atlas tracks; 15 human tissues).
