# Supplementary material for: Long Non-Coding RNA Expression during Aging in the Human Subependymal Zone
Source: Front Neurol. 2015 Mar 9;6:45. doi: 10.3389/fneur.2015.00045 (PMC4353253; doi:10.3389/fneur.2015.00045)
Supplement: Supplementary file 5 [file Image_5.PDF]

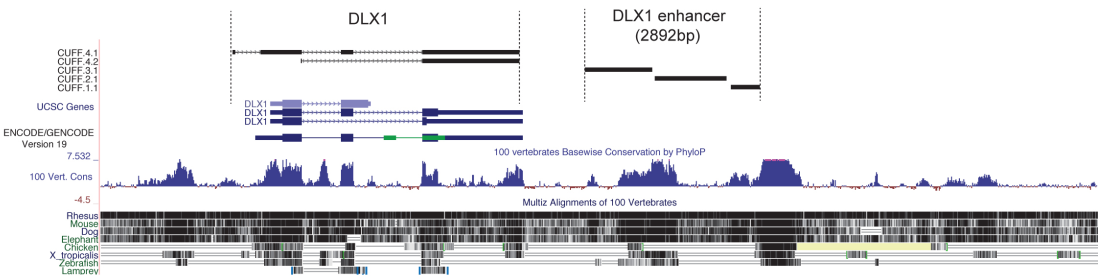

Supplementary Figure 5: Snapshot of the UCSC genome browser (HG19) showing the DLX1 locus and flanking regions. RNA sequencing results (CuffDiff output files; see materials and methods for description) reveal that transcripts relating to the proposed DLX1 enhancer (eDLX1) region are detected in samples from the human subependymal zone. The eDLX1 transcript seems to be well conserved in vertebrates similar to DLX1 (see 100 Vertebrate Conservation track).
